# Editorial: Cryogenic electron microscopy of infectious diseases

**DOI:** 10.3389/fmolb.2024.1506197

**Published:** 2024-10-16

**Authors:** Jiho Yoo

**Affiliations:** College of pharmacy, Chung-Ang University, Seoul, Republic of Korea

**Keywords:** cryogenic electron microscopy, cryo-EM, cryo-ET, infectious disease, protein structure, drug discovery

Cryogenic-electron microscopy (cryo-EM) is becoming a highly helpful technique in the fight against infectious diseases (Renaud et al., 2018; Zhang et al., 2021; Lata et al., 2023). By enabling the atomic-level visualization of the high-resolution structures of biomolecules like viruses and proteins, this technique contributes to our understanding of the precise roles played by pathogens (Jiang and Tang, 2017). It is particularly helping to build medicines and vaccinations that are specifically targeted to the agents that cause infectious diseases (Wibmer et al., 2015; Derking and Sanders, 2021; Yu and Saphire, 2022). This Research Topic on “*Cryogenic Electron Microscopy of Infectious Diseases*” aims to increase knowledge of the applications of cryo-EM, ranging from basic research for infectious agent to the development of medicines against infectious diseases.

In this section, Asarnow et al. emphasized the cryo-electron tomography (cryo-ET) technology in this section in relation to the study of infectious pathogens. Because cryo-ET makes it possible to obtain precise structural information on pathogens—such as viruses, bacteria, and parasites—in their natural habitat, the study of infectious diseases has greatly advanced. This method allows for the monitoring of host-pathogen interactions by providing high-resolution imaging at the subcellular level. The capacity of cryo-ET to analyze these structures without requiring invasive preparation methods provides new insights into the mechanics of bacterial invasion, viral replication, and infection. The accuracy of these insights can be further increased by combining cryo-ET with other techniques such sub tomogram averaging, which could result in advances in therapeutic interventions and drug discovery. Furthermore, the data processing capabilities of cryo-ET are being improved by current advancements in machine learning, enabling quicker and more effective investigation of complex biological systems.

Understanding the structural biology of several infections, such as SARS-CoV-2, the virus that causes COVID-19, has been made possible thanks in large part to cryo-EM. The case of SARS-coV-2’s spike protein, which binds to the ACE2 receptor and allows the virus to enter host cells, was described by Bodakuntla et al. Researchers can now see the spike protein in various conformations thanks to cryo-EM, which has provided vital knowledge about how the virus binds to and infiltrates cells. It has also aided in the identification of the ways in which variations and other changes in the spike protein impact the virus’s ability to evade the immune system and spread. The quick creation of vaccinations and treatment plans has been made possible by these discoveries. Other viral components, such as nonstructural proteins (NSPs), which are involved in viral replication, have also been studied using cryo-EM.

Le et al. further emphasize the structural mechanisms by which SARS-CoV-2 invades host cells. The Spike protein, which binds to the human ACE2 receptor to promote viral entry, is essential to this process. The four main stages of viral entry—prebinding, receptor binding, proteolytic cleavage, and membrane fusion—are separated out in this work. The authors emphasize how the virus can merge with host cell membranes and start an infection due to structural alterations in the Spike protein. Furthermore, Le and colleagues focused on the ways that mutations in the Spike protein, namely, in variations such as Alpha, Beta, Delta, and Omicron, improve the virus’s capacity to attach to ACE2, boost its contagiousness, and occasionally avoid the immune system. They also stress how crucial it is to comprehend these structural alterations for the creation of treatments and vaccines, as structure-based drug development, particularly in light of novel variations, can successfully target particular viral entrance stages.

The impact of cryo-EM on our comprehension of viral structure and infection mechanisms is described by Dutta and Acharya. Researchers can view viruses in their natural states, such as SARS-CoV-2 and HIV-1, at nearly atomic resolution using cryo-EM. The study emphasizes the significance of glycoproteins produced by viruses, such as the coronavirus spike proteins and the HIV Env protein, which are essential for attachment and penetration into host cells. The virus can connect to host receptors and fuse with cell membranes to initiate infection by changing the shape of the spike proteins. The article also explains how Cryo-EM has made it possible to study bacteriophages, or bacterial viruses, and has given researchers new insights into the composition of their capsids, tail fibers, and processes of infection. By offering precise structural details, the high-resolution imaging of cryo-EM aids in the creation of vaccines and antiviral drugs.

Cebi et al. detail how developments in cryo-EM have revolutionized structure-based drug design (SBDD). The study outlines the advantages of the cryo-EM approach in SBDD, such as the capacity to investigate pharmacological targets in conditions similar to their initial state without the need for crystallization. The use of cryo-EM in drug development is made possible by significant technological advancements covered in the study, including improved grid optimization, sample preparation, and data collecting. Recent advances like the merging of molecular dynamics simulations with cryo-EM and machine learning have sped up the process of finding new medications. These advancements enable the speedy identification of drug-binding sites and the synthesis of more effective medicinal molecules.

Overall, we covered a wide range of stories in this Research Topic, from the foundations of the Cryo-EM technique for structural investigations (Asarnow et al.) to the study of pathogen proteins using Cryo-EM and Cryo-ET (Bodakuntla et al., Le et al., Dutta and Acharya) to applications for drug discovery against infectious diseases (Cebi et al.). We anticipate that structural biologists, virologists, and scientists employed in both the public and private sectors will find these tales interesting.
